# In vivo confocal microscopy of pre-Descemet corneal dystrophy associated with X-linked ichthyosis: a case report

**DOI:** 10.1186/s12886-017-0423-5

**Published:** 2017-03-16

**Authors:** Hui Shi, Xiao-feng Qi, Tao-tao Liu, Qian Hao, Xiao-hong Li, Ling-ling Liang, Yi-miao Wang, Zhi-hua Cui

**Affiliations:** Department of Ophthalmology, The First Hospital of Jilin University, No. 71 of xinmin St, Changchun, Jilin Province 130021 China

**Keywords:** Pre-Descemet corneal dystrophy, X-linked ichthyosis, In vivo confocal microscopy, Steroid sulfatase

## Abstract

**Background:**

Pre-Descemet corneal dystrophy (PDCD) is characterized by the presence of numerous, tiny, polymorphic opacities immediately anterior to Descemet membrane, which is a rare form of corneal stromal dystrophy and hard to be diagnosed. In vivo confocal microscopy (IVCM) is a useful tool to examine the minimal lesions of the cornea at the cellular level. In this article, we report a rare case of PDCD associated with X-linked ichthyosis and evaluate IVCM findings.

**Case presentation:**

We present a 34-year-old male Chinese patient with PDCD associated with X-linked ichthyosis. Slit-lamp biomicroscopy showed the presence of tiny and pleomorphic opacities in the posterior stroma immediately anterior to Descemet membrane bilaterally. IVCM revealed regular distributed hyperreflective particles inside the enlarged and activated keratocytes in the posterior stroma. Hyperreflective particles were also observed dispersedly outside the keratocytes in the anterior stroma. Dermatological examination revealed that the skin over the patient’s entire body was dry and coarse, with thickening and scaling of the skin in the extensor side of the extremities. PCR results demonstrated that all ten exons and part flanking sequences of STS gene failed to produce any amplicons in the patient.

**Conclusions:**

IVCM is useful for analyzing the living corneal structural changes in rare corneal dystrophies. We first reported the IVCM characteristics of PDCD associated with X-linked ichthyosis, which was caused by a deletion of the steroid sulfatase (STS) gene, confirmed by gene analysis.

## Background

Pre-Descemet corneal dystrophy (PDCD) is a rare form of corneal dystrophy, characterized by the presence of numerous, tiny, polymorphic opacities in the posterior stroma immediately anterior to Descemet membrane. PDCD commonly occurs in adults aged 30–40 years, and the vision is not usually affected [1, 2]. According to the International Classification of Corneal Dystrophies (IC3D), PDCD is classified into two subtypes: (1) isolated PDCD, with unknown genetic locus; (2) PDCD associated with X-linked ichthyosis, a deletion of steroid sulfatase (STS) gene on chromosome Xp22.3 (MIM #308100) [3].

Ichthyosis is classified into four types: ichthyosis vulgaris (the most common), X-linked ichthyosis, lamellar ichthyosis, and bullous ichthyosiform erythrodermia. X-linked ichthyosis is the second most common form of ichthyosis that commonly affects males with an incidence of 1: 6000 [4]. The disease is early-onset, usually occurring within the first year of the life, and presents with “fish scale” appearance of the skin [5, 6]. X-linked ichthyosis may cause various ocular diseases and PDCD is the most common ocular manifestation [7, 8].

The symptoms and ocular manifestations of PDCD are usually not obvious, therefore it’s difficult for the doctors to diagnose. The use of in vivo confocal microscope (IVCM) makes it possible to observe the different layers of the living cornea at the cellular level and discover the minimal lesions of the cornea. Previous reports have described the characteristics of isolated PDCD using IVCM [2, 9–13]. Here, we present a rare case of PDCD associated with X-linked ichthyosis and evaluate the characteristics of the corneal changes using IVCM.

## Case presentation

**Fig. 1:**
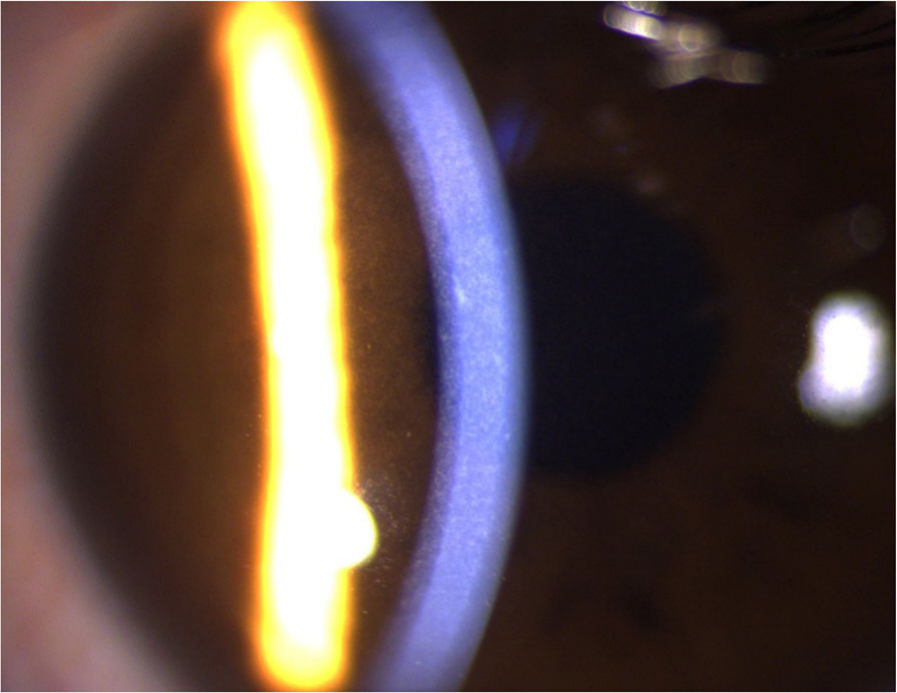
Anterior segment photograph of the right eye. Slit-lamp biomicroscopy showing tiny, pleomorphic, greyish or brownish opacities in the posterior stroma anterior to Descemet membrane

**Fig. 2:**
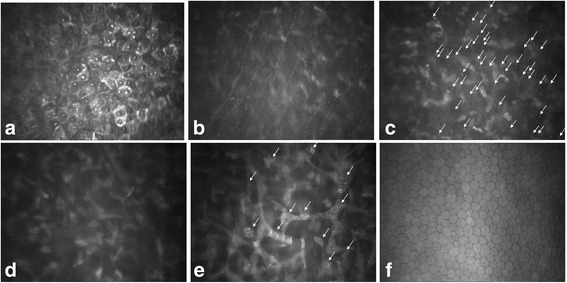
IVCM images of the cornea. **a** Normal appearance of superficial epithelium with prominent nuclei. **b** Normal appearance of parallel subbasal nerves. **c** Anterior stroma with normal-sized keratocytes and extracellular hyperreflective particles. **d** Mid stroma with normal cell density and keratocyte morphology. **e** Posterior stroma with enlarged hyperreflective keratocytes and regularly distributed hyperreflective particles. **f** Normal appearance of the endothelium

**Fig. 3:**
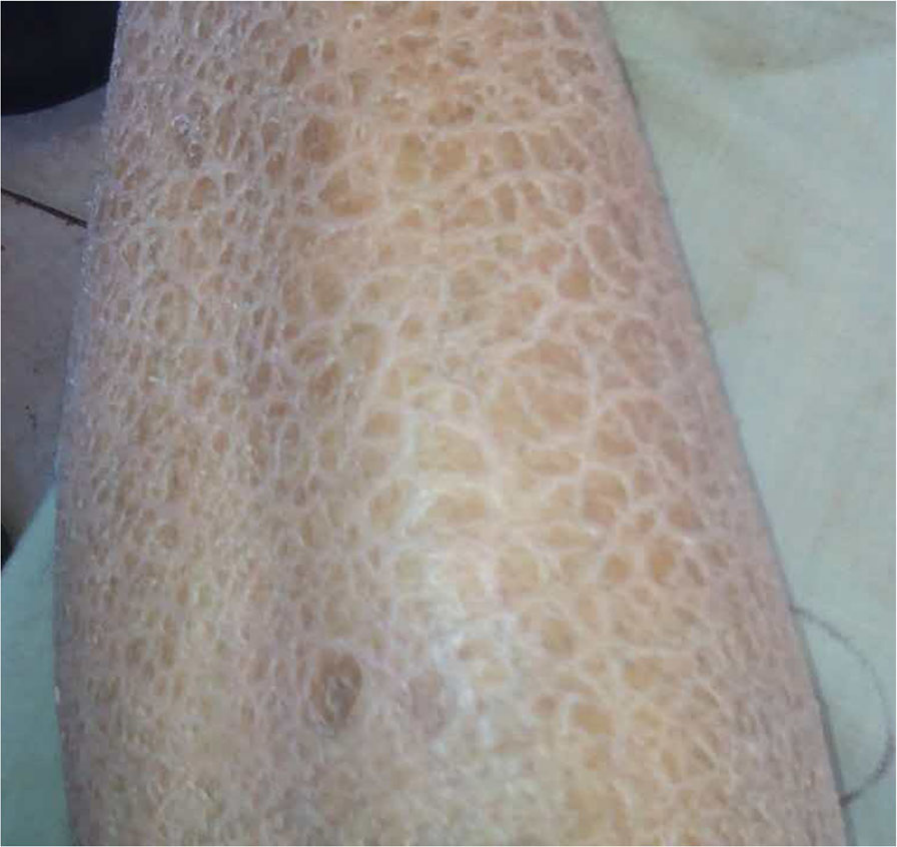
Photograph of the left leg. It showed the thickened “fish scale” appearance of the skin

**Fig. 4:**
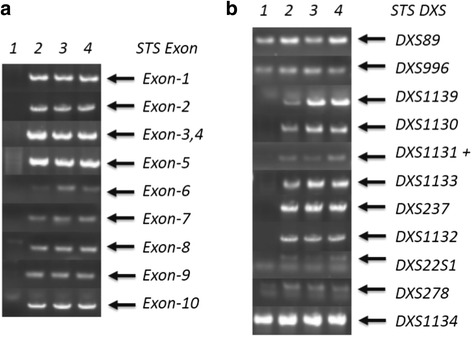
PCR analysis of STS gene. There was a complete deletion in spanning Exon1-Exon10 (**a**) and flanking sequences DXS1139-DXS22S1 (**b**) in the patient,while his father and 2 unrelated healthy volunteers had no such deletion. Lanes 1–4 correspond to the patient, his father and 2 normal controls

A 34-year-old male Chinese patient visited the outpatient department complaining of dry eyes on January 7, 2015. He had a past history of X-linked ichthyosis diagnosed when he was 8 months old. He had no immediate family history of X-linked ichthyosis. But similar ichthyotic skin was found in his maternal grandmother’s brother. The patient had no history of wearing contact lens. The patient had an uncorrected visual acuity of 10/20 in the right eye and 16/20 in the left eye, and had a best corrected visual acuity of 20/20 in both eyes. Refractometric values were found to be -0.50DS–0.75DC × 60 for the right and -1.00DC × 75 for the left eye. Slit-lamp biomicroscopy revealed multiple, tiny, pleomorphic, greyish or brownish opacities anterior to Descemet membrane in the posterior stroma in both eyes. The opacities were diffusely distributed across the cornea, with a 2–3 mm transparent perilimbal zone (Fig. 1). No remarkable abnormalities were found in the anterior segment and the fundus. The corneal thickness was 528 and 522 mm in the right and left eyes, respectively, measured by Oculyzer™. The patient underwent IVCM (ConfoScan 4.0; Nidek Technologies, Albignasego, Italy) to analyze the corneal structure, because of the presence of the corneal stromal deposits. IVCM of the corneal layers revealed a normal epithelium layer. IVCM showed that the cell morphology and density of the keratocytes were normal in the anterior and mid stroma. Multiple hyperreflective particles (2.0–2.5 μm in diameter) were seen dispersedly outside the keratocytes in the anterior stroma (at the depth of 70–164 μm). The enlarged keratocytes were in an activated state with diffuse hyperreflectivity in the posterior stroma (at the depth of 321–494 μm). The polygonal or claw-like shaped keratocytes were interconnected in a net, with regularly distributed hyperreflective particles (2.0–3.4 μm in diameter) inside. The endothelial cells exhibited normal cell morphology with a cell density of 3,347 and 3,095 cells/mm^2^ in the right and left eyes, respectively (Fig. 2). Dermatological examination revealed that the skin all over the body was dry and coarse, with thickening and scaling appearance, predominantly in the extensor side of the extremities (Fig. 3). Polymerase chain reaction (PCR) was used to amplification all the ten exons and the flanking sequences DXS89-DXS1134 of STS gene. The PCR primer design was referred to previous studies [14], and the synthesis was carried out by Comate Bioscience Co., Ltd. After informed consent was obtained, genomic DNA was extracted from the peripheral blood of the patient, his father and two unrelated healthy volunteers. PCR results demonstrated that the patient had a complete deletion in spanning Exon1-Exon10 and flanking sequences DXS1139-DXS22S1 of STS gene,while his father and two unrelated healthy volunteers had no such deletion (Fig 4). The patient was diagnosed with PDCD associated with X-linked ichthyosis. At one year follow-up, visual acuity, slit-lamp biomicroscopy and IVCM did not reveal any detrimental changes in the patient.

## Discussion

As PDCD seldom affects visual acuity and it’s difficult to obtain the corneal tissue, the histopathological study of such corneal dystrophy is rare. Curran et al [15] first performed a histopathological analysis of the cornea from a case of PDCD and found that the keratocytes were enlarged with accumulation of vacuoles in the intracellular compartment containing lipofuscin-like lipoproteins. In an ultrastructural study of a corneal button from a patient with X-linked ichthyosis, Kempster et al [16] found electron-dense polymorphic and lamellated materials along the anterior aspect of Descemet membrane. By contrast, the corneal histopathological features of PDCD and X-linked ichthyosis were similar, and were consistent with the IVCM findings of enlarged keratocytes with intracellular hyperreflective particles in the present case.

In this study, we, for the first time, reported the characteristics of PDCD associated with X-linked ichthyosis using IVCM. We found that the activated keratocytes in the posterior stroma had enlarged cell bodies with a regular arrangement of hyperreflective particles inside. These IVCM findings were similar to the previous reports of isolated PDCD in the literatures [2, 11, 12]. In some other previous studies, the size and morphology of keratocytes in the posterior stroma were reported to be normal in isolated PDCD, although hyperreflective particles were also found in the intracellular and extracellular compartments [9, 10, 13]. The involved corneal layers in PDCD has been reported to be mainly limited within the posterior stroma immediately anterior to Descement membrane as observed by slit-lamp biomicroscopy, and confirmed by Malhotra et al [2] using anterior segment optical coherence tomography. Similarly, some IVCM studies have revealed that the lesion of PDCD was restricted within the posterior stroma [9–13]. However, other IVCM studies have found that the lesion of PDCD was involved in the whole thickness of the corneal stroma [9, 10, 13]. The involvement of corneal endothelial layer was also reported in some PDCD patients [2, 12]. In our present case, using IVCM, we found that the enlarged keratocytes with hyperreflective particles in the posterior stroma were consistent with the histopathological study. We also found hyperreflective particles outside the anterior stroma, which has not been reported previously.

X-linked ichthyosis is a genetic disorder of the skin caused by mutation or deletion of the STS gene on chromosome Xp22.3. The STS gene is composed of 10 exons that span a region of about 140 kb. Up to 90% X-linked ichthyosis patients exhibit large deletions of the entire STS gene and flanking sequences, while a minority show a point mutation or partial deletion of the STS gene [17, 18]. In our case, we demonstrated that the patient had a deletion of the entire STS gene and flanking sequences DXS1139-DXS22S1 using PCR. He had no immediate family history of X-linked ichthyosis, but similar ichthyotic skin was found in his maternal grandmother’s brother. So we speculated that his mother and his maternal mother were heterozygous females, and the STS gene deletion of our patient was hesitated from them. Hung et al [19] also demonstrated that the corneal changes in PDCD and X-linked ichthyosis were associated with deletion of the STS detected with microarray-based comparative genomic hybridization. STS is found throughout the body, including the epidermis, where it is thought to play a role in the steroid production and lipid regulation of the stratum corneum [20]. As STS deficiency leads to elevated plasma levels of cholesterol sulfate, the characteristic feature of posterior stromal opacities in PDCD has been postulated to represent focal accumulations of cholesterol sulfate [7, 16]. Therefore, we propose that STS deficiency may lead to lysosomal dysfunction and lipid metabolism disorder, thus leading to accumulation of undigested substances in the intracellular and extracellular compartments of keratocytes. This may explain the hyperreflective particles in the anterior and posterior stroma identified in our patient by IVCM.

## Conclusions

In summary, we first reported the IVCM characteristics of PDCD associated with X-linked ichthyosis. These IVCM findings of PDCD may be associated with STS deficiency, caused by X-linked ichthyosis. Gene analysis demonstrated that there was complete a deletion of all ten exons and part flanking sequences of STS gene in the patient. Therefore, IVCM is useful for analyzing the corneal structural changes in rare corneal dystrophies where availability of corneal tissues is limited for examination.

## Abbreviations

IC3D: The International classification of corneal dystrophies
IVCM: In vivo confocal microscopy
PDCD: Pre-descemet corneal dystrophy
STS: Steroid sulfatase
